# Designing a new National Linkage Model to bring together data across Australian agencies and jurisdictions to deliver the National Disability Data Asset (NDDA) and establish infrastructure for the delivery of future linked assets

**DOI:** 10.23889/ijpds.v9i5.2819

**Published:** 2024-09-18

**Authors:** Theresa Nunan, Leigh Hastwell, Tammy-Lee Purssell, Bronwyn Mury

**Affiliations:** 1 Australian Bureau of Statistics

The National Disability Data Asset (NDDA) will bring together data across jurisdictions to provide a more complete picture of the life experiences of people with disability. The National Linkage Model (NLM) describes a new approach within the Australian data linkage landscape for bringing data together across agencies and jurisdictions, drawing upon expertise from Commonwealth, State and Territory population data centres to collaboratively deliver the linked data asset. The NLM will enable delivery of the NDDA, while also establishing enduring, reusable infrastructure to efficiently deliver future national and jurisdictional data assets.

As Australia does not have a national identifier, a National Linkage Spine (NLS) is created from a group of core datasets to act as a secure, restricted-access conduit for connecting data across jurisdictions. Data is securely linked to the NLS by Accredited Data Service Providers (ADSPs), with the results fed into the centrally managed National Linkage Map (NLMap). The NLMap is then used to collate de-identified analytical data from disparate datasets across jurisdictions to build the NDDA (or other approved linked assets) whilst ensuring critical privacy protections are met.

The NLM will enable person level data to be bought together on a scale not previously seen in Australia. However, the collaborative approach to building the asset introduces new complexities such as managing version control across environments and measuring quality when multiple linkage methods have been used. This innovative, collaborative, cross-jurisdictional approach will support high-quality analysis of life experiences of people with disability, and establish a reusable approach for future assets.

